# Excretable, ultrasmall hexagonal NaGdF_4_:Yb50% nanoparticles for bimodal imaging and radiosensitization

**DOI:** 10.1186/s12645-021-00075-x

**Published:** 2021-02-05

**Authors:** Jossana A. Damasco, Tymish Y. Ohulchanskyy, Supriya Mahajan, Guanying Chen, Ajay Singh, Hilliard L. Kutscher, Haoyuan Huang, Steven G. Turowski, Joseph A. Spernyak, Anurag K. Singh, Jonathan F. Lovell, Mukund Seshadri, Paras N. Prasad

**Affiliations:** 1 Department of Chemistry and Institute for Lasers, Photonics and Biophotonics, University At Buffalo, The State University of New York, Buffalo, NY 14260 USA; 2 Department of Medicine, Division of Allergy, Immunology and Rheumatology, University At Buffalo, The State University of New York, Buffalo, NY 14203 USA; 3 Department of Biomedical Engineering, University At Buffalo, The State University of New York, Buffalo, NY 14260 USA; 4 Translational Imaging Shared Resource, Roswell Park Comprehensive Cancer Center, Buffalo, NY 14263 USA; 5 Department of Radiation Medicine, Roswell Park Comprehensive Cancer Center, Buffalo, NY 14263 USA; 6 College of Optoelectronic Engineering, College of Physics and Optoelectronic Engineering, Shenzhen University, 518060 Shenzhen, People’s Republic of China; 7 Department of Anesthesiology, University At Buffalo, The State University of New York, Buffalo, NY 14214 USA; 8 Department of Oral Oncology/Dentistry and Maxillofacial Prosthetics, Roswell Park Comprehensive Cancer Center, Buffalo, NY 14263 USA; 9 School of Chemistry and Chemical Engineering, Harbin Institute of Technology, Harbin, Heilongjiang 15001 People’s Republic of China; 10 Department of Interventional Radiology, The University of Texas MD Anderson Cancer Center, Houston, TX 77030 USA

**Keywords:** Gadolinium nanoparticles, Radiosensitizer, Theranostics, MR/CT imaging probes, Glioblastoma

## Abstract

**Background:**

In this study, we report on the synthesis, imaging, and radiosensitizing properties of ultrasmall β-NaGdF_4_:Yb50% nanoparticles as a multifunctional theranostic platform. The synthesized nanoparticles act as potent bimodal contrast agents with superior imaging properties compared to existing agents used for magnetic resonance imaging (MRI) and computed tomography (CT). Clonogenic assays demonstrated that these nanoparticles can act as effective radiosensitizers, provided that the nanoparticles are taken up intracellularly.

**Results:**

Our ultrasmall β-NaGdF_4_:Yb50% nanoparticles demonstrate improvement in T1-weighted contrast over the standard clinical MR imaging agent Gd-DTPA and similar CT signal enhancement capabilities as commercial agent iohexol. A 2 Gy dose of X-ray induced ~ 20% decrease in colony survival when C6 rat glial cells were incubated with non-targeted nanoparticles (NaGdF_4_:Yb50%), whereas the same X-ray dose resulted in a ~ 60% decrease in colony survival with targeted nanoparticles conjugated to folic acid (NaGdF_4_:Yb50%-FA). Intravenous administration of nanoparticles resulted in clearance through urine and feces within a short duration, based on the ex vivo analysis of Gd^3+^ ions via ICP-MS.

**Conclusion:**

These biocompatible and in vivo clearable ultrasmall NaGdF_4_:Yb50% are promising candidates for further evaluation in image-guided radiotherapy applications.

## Background

Clinically relevant, multifunctional nanoparticles that combine diagnostic and therapeutic platforms are of high scientific interest, with significant societal impact (Chen et al. 2016,2017; Dasgupta et al. 2020). However, these theranostic nanomaterials often result in complex and large-sized structures to accommodate the various components that provide multi-functionality. In addition, complicated synthesis methods are difficult to reproduce and can be impractical for large-scale processing.

To be clinically relevant, nanomaterials need to exhibit biocompatibility and ideally undergo rapid clearance from the body. (Yang et al. 2019) Nanoparticles are primarily taken up and eventually cleared by the reticuloendothelial system (RES) or kidneys. However, retention in the liver or spleen can take up a long time depending on their size and surface chemistry. (Kermanizadeh et al. 2020; Feliu et al. 2016) These accumulations in the RES and the slow elimination, taking months or even years to clear the body, can be problematic for clinical translation. On the other hand, clearance through the kidneys occurs quickly, as the nanoparticles are filtered from the blood and excreted out. This significantly reduces the risk of potential toxicity, which makes the renal clearance pathway an attractive route of elimination. (Du et al. (2018)) However, glomerular filtration is strongly dependent on size, with a hydrodynamic diameter filtration-size threshold of < 6 nm. (Longmire et al. 2008) Thus, the development of ultrasmall, biocompatible, multifunctional nanoparticles is of high interest for potential clinical use (Longmire et al. 2008; Xie et al. 2020; Choi et al. 2010).

One of the promising applications of theranostic nanoparticles is their ability to enhance radiotherapeutic efficacy. Radiation therapy (RT) is an integral part of clinical management of most solid tumors, and remains one of the most cost-effective treatments for cancer patients (Retif et al. 2015). However, not all patients respond to RT, and disease recurrence remains a significant clinical problem (Platek et al. 2013).

The use of nanoparticles to ‘sensitize’ tumors to RT could potentially enable lowering of the total radiation dose administered to patients, without compromising efficacy. Furthermore, radiation-induced normal tissue toxicity often contributes to a poor quality of life (QOL) in patients. Optimal use of these nanoparticles in combination with RT may therefore minimize collateral radiation damage to normal tissues and potentially improve QOL.

In this regard, there is growing interest in the use of metal nanoparticles as radiosensitizers. Upon irradiation, metal nanoparticles, with their high surface area and surface chemistries, have shown intrinsic radiocatalytic activities in water producing reactive oxygen species (ROS), thus, effectively increasing the overall radical concentration. (Guerreiro et al. 2019) Furthermore, interactions of X-ray with metals with high atomic number (high Z) are known to enhance the photoelectric and Compton effects that result in radiation dose-enhancements.(Retif et al. (2015)) Most studies have focused on gold (*Z* = 79) nanoparticles and significant evidences have been reported to demonstrate their ability to increase the therapeutic ratio of radiotherapy. (Penninckx et al. (2020); Laprise-Pelletier et al. 2018; Ngwa et al. 2014; Babaei and Ganjalikhani 2014) Hafnium oxide (*Z* = 72) developed by NanoBiotix (France) has already shown success in the clinic as efficient radiation enhancers on patients requiring preoperative radiotherapy.(Bonvalot et al. 2019) Other metal nanoparticles that have shown great promise in augmenting radiotherapy include bismuth (*Z* = 83) (Deng et al. 2018), platinum (*Z* = 78) (Erika et al. 2010; Li et al. 2019), and iron oxide (Klein et al. 2012; Khoei et al. 2014) having both radiosensitizing and hyperthermic (Cassim et al. 2009) properties.

Both gadolinium-based (*Z* = 64) and ytterbium-based (*Z* = 70) nanoparticles have garnered attention as theranostic platforms. Gd-based nanoparticles have been shown to enhance magnetic resonance (MR) imaging contrast and have been found to induce X-ray dose enhancement**,** making them ideal candidates for combined imaging and therapy, and are currently in Phase I clinical trial for the treatment of multiple brain metastases.(Dufort et al. 2019; Duc et al. 2014; Verry et al. 2019) Yb-based nanoparticles have been developed as bimodal probes for X-ray computed tomography (CT) and near infrared-to-near infrared fluorescence imaging. (Xing et al. 2012) The high X-ray attenuation of Yb enabled its use as a theranostic agent, with both tumor imaging and radiosensitization functions. (Xing et al. 2013).

In this study, we present nanoparticles containing Gd and Yb as candidates for combined imaging and therapy in a single ultrasmall nanoplatform for cancer therapy. The combination of Gd and Yb allows for the nanocrystal serving as a bimodal imaging probe for MR and CT examinations. MR imaging is best suited for soft-tissue imaging, while X-ray CT is ideal for hard tissues or bone. In addition, reducing the size of the nanoparticles to sub-5 nm increases the surface Gd^3+^ accessible to H_2_O which leads to higher T1 relaxivities in comparison to larger nanoparticles,(Johnson et al. 2011) while allowing complete elimination from the body within days (i.e., 4 days) through hepatic and renal clearance, as revealed from our ICP-MS analysis of Gd^3+^. The first reported use of combined Gd and Yb as an MR/CT probe was in the form of NaGdF_4_:Yb20% doped with 2% Erbium(Er) for additional optical imaging capability. (He et al. 2011) In addition, the CT signal was enhanced by increasing the amount of Yb from 20 to 80% (i.e., NaYbF_4_:Gd20%). (Liu et al. 2012) However, for obtaining the preferred thermodynamically stable hexagonal phase, both these preparations resulted in large-sized nanoparticles (i.e. > 20 nm). In addition, prior to the synthesis of the nanocrystals, an initial step to prepare the lanthanide precursors is needed. Here, we present a more practical and user-friendly approach introducing a single-step method that allows a precise control of size, uniformity, and crystal phase. Because MRI is the more sensitive modality, it is ideal to have a higher ratio of Yb to Gd which would result in much higher X-ray attenuation. On the other hand, increasing the concentration of Yb generally produces larger nanoparticles. (He et al. 2011; Liu et al. 2012; Damasco et al. 2014) Thus, to guarantee that the ultrasmall size is maintained in the hexagonal phase, our nanoparticles were designed with an equimolar amount of Gd^3+^ and Yb^3+^ ions.

To ensure effective radiosensitization, these nanoparticles were modified for targeted delivery by conjugating folic acid to their surface, ensuring optimal cellular uptake by cancer cells, which ultimately significantly decreased the number of surviving colonies following a clinically relevant X-ray exposure. Ultra-small size nanoparticles are also ideal for radiotherapy due to low self-absorption of electrons resulting in higher Auger electron yield. (Hossain and Su 2012) These emitted secondary electrons have very low energy and thus short-range to produce localized cellular damage.

We evaluated the efficacy of this nanoplatform in an in vitro clonogenic assay using C6 rat glioblastoma cells. Glioblastoma multiforme (GBM) is a grade IV tumor and represents about 15% of all primary brain tumors. It is the most aggressive and infiltrative form of gliomas, quickly spreading in all parts of the brain (Holland 2000) The average survival for GBM is 12–15 months using the current standard of care treatment, and the determination of treatment response and clinical decision-making are based on the accuracy of radiographic assessment. (Arvold and Reardon 2014) A major factor that contributes to poor prognosis in GBM patients is the limited response to treatment caused by the inability of most chemotherapeutic agents to cross the blood–brain barrier (BBB). We demonstrate here that the ultrasmall size of the nanoparticles conjugated with folic acid can take advantage of the folate receptor expressed at the BBB (Afzalipour et al. 2019; Wu and Pardridge 1999) to facilitate the transport of the nanoparticles across BBB. Advanced MRI and CT imaging techniques such as dynamic contrast-enhanced (DCE) imaging, which monitors the temporal changes in contrast enhancement in blood vessels and tissues to provide a time-concentration curve, are promising non-invasive methods with moderate-to-high accuracy in stratifying tumors and discriminating recurrent lesions and treatment-related changes. (Abdel Razek et al. 2015,2017; O'Connor et al. 2011; Okuchi et al. 2019) Given the marked signal enhancement on both MR and CT imaging, our nanoparticles could enable accurate diagnosis of disease progression of GBM.

With its facile synthesis, highly uniform size distribution, ultrasmall size and easily tailored surface, these novel nanoparticles present a promising, translatable theranostic platform with high tumor uptake, favorable biodistribution, and route of elimination.

## Results

### ***Formation of ultrasmall β-NaGdF***_***4***_***:Yb50%***

**Fig. 1:**
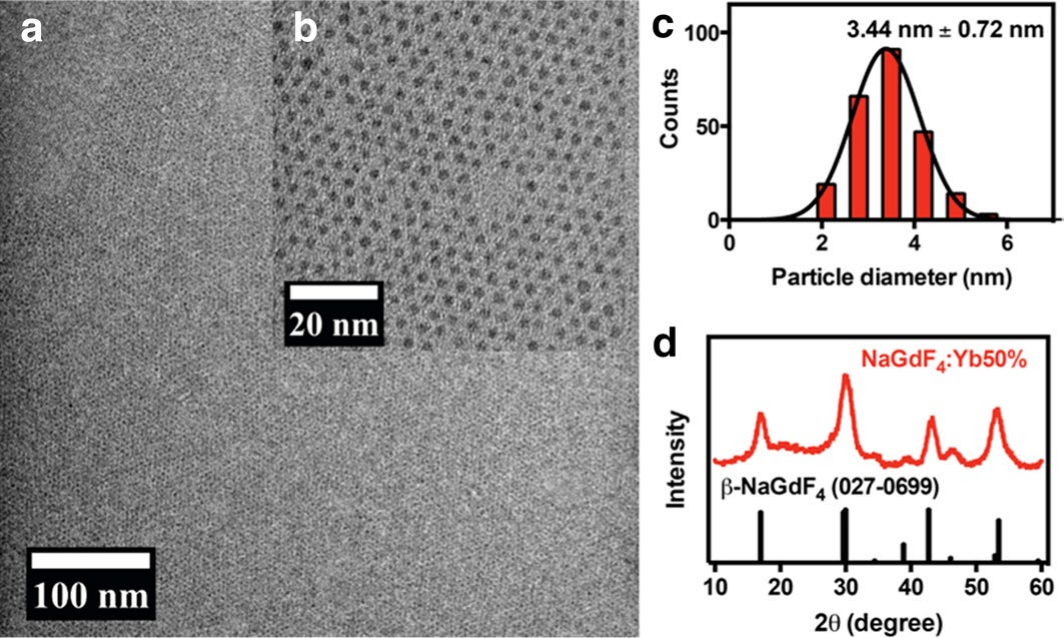
Characterization of β-NaGdF_4_:Yb50%. **a** TEM and **b** HRTEM images show that the synthesized nanoparticles are uniform and monodisperse with a core diameter less than 5 nm; **c** size distribution determined from several TEM images. **d** XRD reveals the hexagonal crystal structure, which is the thermodynamically stable phase of the nanocrystal

Uniform sub-5 nm NaGdF_4_:Yb50% nanoparticles in a thermodynamically stable, hexagonal phase (β-phase) were successfully synthesized. Analysis of more than 100 nanoparticles from TEM images reveals a normal size distribution with an average diameter of 3.44 nm ± 0.72 nm (Fig. 1a–c). The formation of ultrasmall β-NaGdF_4_:Yb50% nanoparticles is confirmed by its very broad X-ray diffraction patterns, which conform to the standard XRD peaks of the hexagonal β-phase NaGdF_4_ (JCPDS 27–0699) (Fig. 1d). Elemental analysis of Gd and Yb content shows the respective actual molar percentages to be 52.18% and 47.82%, a clear indication that the desired stoichiometric amount of Yb^3+^ ions was successfully doped into the NaGdF_4_ nanoparticle.

**Fig. 2:**
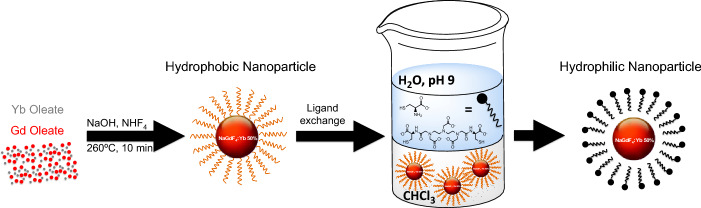
Schematic illustration of the synthesis and surface modification of ultrasmall oleic acid-stabilized NaGdF_4_:Yb50% nanoparticles

NaREF_4_ (RE = rare earth) nanoparticles are known to exist in two phases, the metastable cubic α-phase and the thermodynamically stable hexagonal β-phase (Mai et al. 2006). This difference in stability has been exploited in the focusing of particle size distribution, wherein the more soluble α-phase nanoparticles serve as sacrificial precursors to form the thermodynamically preferred β-phase with narrow distribution (Dühnen et al. 2015; Naduviledathu Raj et al. 2014; Rinkel et al. 2014). This method typically results in larger nanoparticles, although Haase et al. have successfully synthesized 5.6 nm β-NaYF_4_:Yb 20%, Er 2% nanoparticles by heating 10 nm sacrificial α-NaYF_4_:Yb 20%, Er 2% (Rinkel et al. 2014). However, a more practical and user-friendly approach is to have a single-step method that will allow precise control of size, uniformity, and crystal phase (Fig. 2).

Surface modification of the nanoparticle surface was achieved through ligand exchange by allowing the nanoparticles in chloroform solution, and the L-cysteine and DTPA anhydride in basic water (pH 9) to mix for 24 h. The hydrodynamic diameter measured by dynamic light scattering (DLS) showed an increase in the hydration shell from 4.1 nm (in hexane) to 5.1 nm (in H_2_O) (Additional file 1: Figure S1) after surface modification. TEM images did not show clustering or aggregation of the nanoparticles suspended in H_2_O (Additional file 1: Figure S2). This successful coating of the ligands on the nanoparticle surface also provided additional functional groups (i.e., amine and carboxylate) to allow bioconjugation of targeting ligands.

### ***Gd***^***3***+^***leaching, cytotoxicity, and biodistribution***

**Fig. 3:**
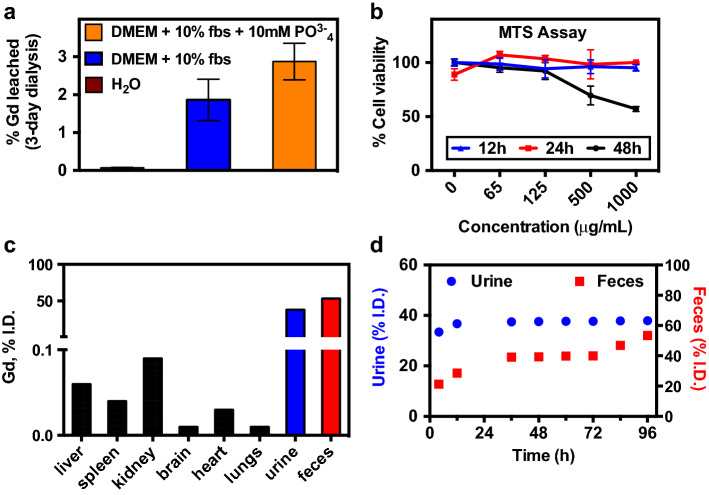
Biocompatibility of H_2_O-dispersed β-NaGdF_4_:Yb50%: **a** ICP-OES analysis of the Gd^3+^ ions leaching from the nanoparticles after 3 days of dialysis. **b** Cell viability when incubated with the nanoparticles evaluated by the MTS assay. **c** Biodistribution at 4 h post-injection via tail vein measured by ICP-MS. **d** Cumulative renal and fecal clearance of the nanoparticles monitored by Gd^3+^ determination via ICP-MS

The stability of NaGdF_4_ was evaluated by measuring the Gd^3+^ ion leakage from the crystal matrix. An analysis of Gd^3+^ leaching shows less than 0.1% Gd^3+^ ions were present when dialyzed against H_2_O. Solutions of DMEM with 10% FBS, and DMEM with 10% FBS supplemented with 10 mM phosphate, incubated at 37 °C, were utilized to mimic physiological conditions and to assess the effect of elevated phosphate levels on the stability of the nanoparticles. After 3 days of dialysis, ~ 2% of the Gd^3+^ was observed in dialysate; this rose to ~ 3% at higher phosphate concentrations (Fig. 3a).

The effect of the nanoparticles on cell viability was studied by monitoring the mitochondrial metabolic activity through the standard MTS assay. C6 cells remained 100% viable after 12 and 24 h incubation at up to 1 mg/mL (Fig. 3b). More importantly, cells remained 100% viable even with increased incubation time (48 h), at 125 μg/mL. It is generally accepted that nanoparticle toxicity is concentration- and time-dependent (Kong et al. 2011; Lewinski et al. 2008). Similarly, further increase in the concentration of the nanoparticles to 1 mg/mL at prolonged exposure time (i.e., 48 h) resulted in increased cytotoxicity (50% of cell viability).

Passive biodistribution and clearance studies revealed that less than 0.5% of the nanoparticles remained in the organs after 4 days, as detected by ICP-MS (Fig. 3c). After 4 h, 33% of the nanoparticles were eliminated through the urine and 21% through the feces (Fig. 3d). The remaining nanoparticles were eliminated mostly through the feces over a period of 4 days (Fig. 3d).

### Nanoparticles for MRI and CT imaging

**Fig. 4:**
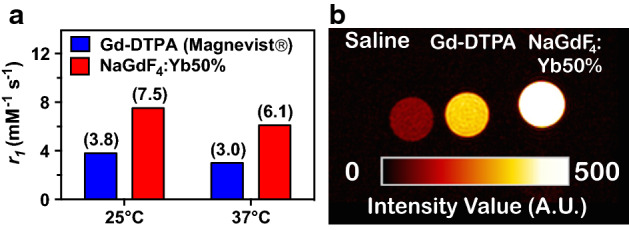
**a** Comparison of in vitro, longitudinal relaxivity values (r1), at 4.7 T for commercially available Gd-DTPA vs ultrasmall NaGdF_4_:Yb50% at 25 °C and 37 °C. **b** Pseudo-colored, T1-weighted MR image for saline, Gd-DTPA, and ultrasmall NaGdF_4_:Yb50%. Ultrasmall nanoparticles and Gd-DTPA samples contain the same concentration of Gd^3+^ (200 μM).

The potential of these nanoparticles as a bimodal imaging probe for both MR and CT imaging was evaluated by measuring their T1 relaxivity (*r1*) and the Hounsfield unit (HU) values, respectively. The relaxivity of the nanoparticles was compared to Gd-DTPA (Magnevist®) at 25 °C and 37 °C by measuring T1 rates of a series of solutions containing increasing Gd^3+^ molar concentrations (as determined by ICP-OES). There is a linear relationship between the Gd^3+^ concentration and the longitudinal relaxation rate (1/T1), and *r1* values are determined from the slope of the resulting linear plots (Additional file 1: Figure S3). A pseudo-colorized, T1-weighted spin echo image (TE/TR = 8.5/500 ms) for saline, 200 μM Gd-DTPA, and nanoparticles (200 μM [Gd]) demonstrates the improvement in T1-weighted contrast of the nanoparticles over the standard clinical MR imaging agent Gd-DTPA (Fig. 4b).

**Fig. 5:**
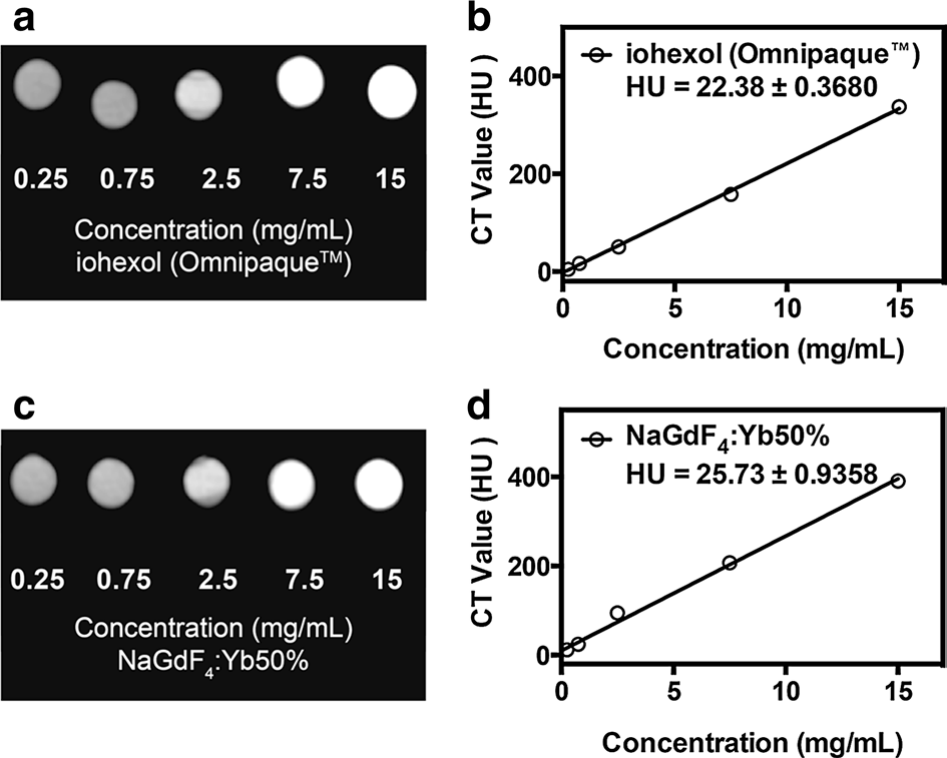
**a** and **c** CT images and the respective **b** and **d** HU measurements of iohexol and NaGdF_4_:Yb50% nanoparticles at different concentrations in H_2_O.

The Hounsfield unit (HU) value, determined from the slope of the linear plot of HU as a function of the concentration, can indicate if the nanoparticles can serve as a CT contrast agent. There is a linear correlation between the increasing contrast agent concentration and the CT signal intensity for both the commercial agent iohexol and the nanoparticle solution (Fig. 5a and c). The nanoparticles and iohexol show almost identical line slopes (Fig. 5b and d) indicating similar signal enhancement capabilities. Setting the HU value of water as zero, the calculated slope for the HU value for the ultrasmall NaGdF_4_:Yb50% is approximately 26 HU while that of the iohexol is about 23 HU.

### Nanoparticles as a radiosensitizer

**Fig. 6:**
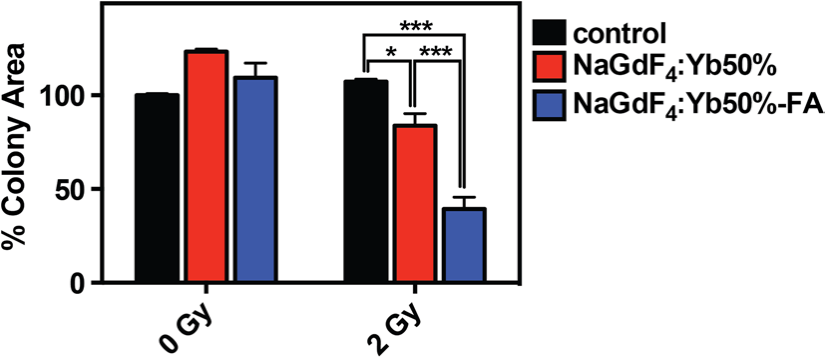
Effect of the nanoparticle treatment on the colony formation of C6 cells following 2 Gy X-ray irradiation. The cells were incubated with the nanoparticles overnight prior to irradiation. The surviving fraction for each treatment was tested using two-way ANOVA with Tukey’s multiple comparisons test. (*P < 0.05; *** *P* < 0.001)

A clonogenic assay was used to investigate the potential of the nanoparticles as radiosensitizers in a rat C6 glioma cell line. To ensure that cell death was not due to any inherent toxicity of the nanoparticles, the concentration was kept at 100 μg/mL, which still maintained more than 90% cell viability even after 48 h incubation (Fig. 3b). Colony formation of the cells without nanoparticles and without X-ray radiation treatment served as control. C6 cells incubated with nanoparticles but not subjected to X-ray radiation did not reduce surviving colonies, confirming that the nanoparticle concentration was not cytotoxic (Fig. 6). Irradiation alone of the cells with a 2 Gy dose did not result in any significant cell reproductive death. Cells treated with non-targeted NaGdF_4_:Yb50% nanoparticles showed a 16% decrease in surviving colonies, in comparison to cells treated only with X-ray radiation. In comparison, targeted NaGdF_4_:Yb50%-FA nanoparticles demonstrated superior efficacy with only 40% surviving colonies when treated with 2 Gy radiation (Fig. 6).

### Nanoparticles cross the blood–brain barrier

**Fig. 7:**
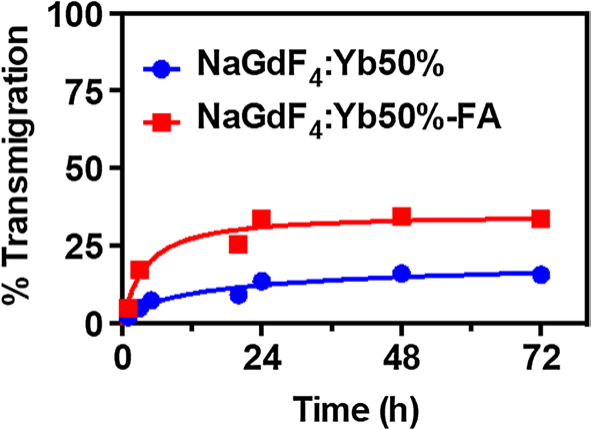
Transmigration of nanoparticles across the in vitro BBB

To further test the potential application of these ultrasmall nanoparticles to treat brain tumors, the ability to cross the blood–brain barrier (BBB) was explored utilizing a previously reported cell-based two-chamber in vitro transwell model of the BBB (Mahajan et al. 2008; Singh et al. 2016). Both non-targeted and FA-targeted nanoparticles demonstrate the ability to cross the BBB (Fig. 7). After 3 h, only ~ 5% of the non-targeted NaGdF_4_:Yb50% nanoparticles crossed the BBB, whereas ~ 17% of the targeted NaGdF_4_:Yb50%-FA crossed. The rate of cell uptake was very gradual for the non-targeted NaGdF_4_:Yb50% nanoparticles, and only ~ 14% were able to cross in 24 h. The targeted NaGdF_4_:Yb50%-FA nanoparticles saturated uptake at 24 h and ~ 34% of the nanoparticles were able to cross BBB at 24 h. Both the non-targeted and targeted nanoparticle uptake had little further uptake between 24 and 72 h (Fig. 7).

## Discussion

To produce uniform ultrasmall size nanoparticles utilizing a single-step method, it is critical that sufficient nucleation occurs to ensure uniformity, and the reaction temperature (e.g., 270 °C) is reduced to decrease the particle size (Johnson et al. 2011; Jin et al. 2015; Xing et al. 2014). It is well established that the hexagonal β-phase NaGdF_4_ nanoparticles readily form at reaction temperatures below 300 °C (Johnson et al. 2011; Jin et al. 2015). This is due to the large radius of the light lanthanide Gd^3+^ ion that is more polarizable and susceptible to the electron cloud distortion required for the cubic-to-hexagonal-phase transformation (Damasco et al. 2014; Noculak et al. 2015; Wang et al. 2010). However, incorporation of the smaller Yb^3+^ ions into the NaGdF_4_ nanoparticles resulted in an increased free-energy barrier with regards to the formation of the hexagonal phase nanoparticles. Thus, significant doping of the Yb^3+^ ion into the host lattice favors the formation of the cubic phase nanoparticles, which are easily produced due to the high surface energy of the ultrasmall nanoparticles. This is in agreement with the results of our synthesis of pure NaGdF_4_, pure NaYbF_4_, and NaGdF_4_:Yb50% nanoparticles (Additional file 1: Figure S4). Allowing nucleation at room temperature for 30 min and subsequently growing the nanoparticles at 260 °C for 10 min yielded hexagonal NaGdF_4_, while both pure NaYbF_4_ and NaGdF_4_:Yb50% resulted in cubic phase nanoparticles as evidenced by their respective XRD patterns (Additional file 1: Figure S1). One way to achieve hexagonal β-NaGdF_4_:Yb50% is to increase the temperature to 300 °C, but this also leads to formation of larger nanoparticles (~ 12 nm) (Damasco et al. 2014). Hence, to form hexagonal NaGdF_4_:Yb50%, nucleation and growth are allowed to take place for 24 h to facilitate the formation of thermodynamically stable, hexagonal nanocrystals, while still maintaining the nanoparticle growth reaction temperature at 260 °C for 10 min to tune the size of the nanoparticles. Pure NaYbF_4_ was also synthesized with 24 h nucleation to check if β-NaYbF_4_ can form under such conditions. The XRD pattern (Additional file 1: Figure S5) revealed a pure cubic α-phase, indicating that the reaction conditions were not sufficient to transform to hexagonal NaYbF_4_. Cubic nanoparticle formation was expected, since the formulation did not contain Gd^3+^ ions, which have been established to lower the energy barrier for phase transformation of NaYbF_4_ (Damasco et al. 2014).

To render the β-NaGdF_4_:Yb50% nanoparticles useful for biological applications, it is necessary to modify the hydrophobic oleic-capped surface with a biocompatible, hydrophilic ligand. The proximity of water protons to the surface of the nanoparticles is critical in achieving high T1 relaxivity, which can be controlled through a surface coating strategy.(Johnson et al. 2016) Phase transfer via ligand exchange was then performed to ensure efficient surface hydration. Removal of oleic acid avoids the formation of long hydrophobic chains that could render the Gd on the surface of the nanoparticles inaccessible to water. (Fang et al. 2014) In this case, cysteine-DTPA replaced oleic acid on the surface of the nanoparticles to form a stable monodisperse aqueous suspension. The small increase in the hydrodynamic diameter post-surface modification indicates the formation of a compact hydrophilic surface.

The potential toxicity of the non-targeted nanoparticles was investigated to assess their practical usability in a biological environment. One major challenge in the development of a Gd-based contrast agent is the inherent toxicity of the Gd^3+^ ion when dissociated from its chelate in vivo.(Zhou and Lu 2013) In the nanocrystal form (i.e., NaGdF_4_), the hexagonal phase provides a stable matrix that eliminates transmetallation with endogenous metal ions (i.e., Cu^2+^, Zn^2+^, Fe^2+^/Fe^3+^) (Morcos 2008; Rabiet et al. 2014; Telgmann et al. 2012; Wu et al. 2010) and hinders any leaching of toxic, free Gd^3+^ ions. (Chen et al. 2011; Kumar et al. 2009) The very low concentration of Gd^3+^ when dialyzed against H_2_O demonstrates the high stability of the nanoparticles against dissolution attributed to their thermodynamically stable hexagonal phase. (Lisjak et al. 2015) However, the presence of elevated phosphate levels resulted in a significant increase in leakage, although still a low percentage of Gd^3+^, indicating the stability of the nanoparticles in a physiological environment.

It has been demonstrated that the capping ligand has stabilizing effects and can sequester the free Gd^3+^ ions through chelation.(Xing et al. 2014; Ahrén et al. 2010; Mekuria et al. 2017) To further investigate and minimize the Gd^3+^ leakage, two strategies could be pursued to improve the design of the surface ligand in relation to Gd^3+^ release. First, the amount of DTPA conjugated to cysteine could be optimized. Second, DTPA can be replaced with other polyaminocarboxylate ligands such as 1,4,7,10-tetraazacyclododecane-1,4,7,10-tetraacetic acid (DOTA) and derivatives, which are known to form lanthanide complexes with high kinetic stability (Tei et al. 2015; Zhu and Lever 2002).

No intrinsic cytotoxicity from the nanoparticles was observed at a concentration as high as 125 μg/mL, even at prolonged exposure time (i.e., 48 h). In vivo clearance study show that the nanoparticles are cleared from the body within days (i.e., 4 days). Furthermore, the fact that the nanoparticles can be cleared through hepatobiliary excretion indicates a decrease in kidney load compared to commercially available Gd^3+^ chelates for MRI (i.e., Gd-DTPA) (Yu and Zheng 2015) which are primarily cleared renally. This can potentially avoid contrast-induced nephropathy, a form of acute renal failure caused by exposure to the contrast media, and may lower the risk for developing nephrogenic systemic fibrosis, triggered in patients with advanced kidney disease.(Perazella 2009).

After establishing the biocompatibility of the nanoparticles, their ability to be used for dual MR/CT imaging was verified. In vitro experiments revealed a substantially higher T1 relaxivity of the nanoparticles compared to a commercial Gd^3+^ chelate at both room temperature and at physiological temperature (37 °C) (Fig. 4a), which may be attributed to the slower tumbling rate of the nanoparticle than the chelate. (Hou et al. 2013) The higher T1 relaxivity values exhibited by the ultrasmall NaGdF_4_:Yb50% nanoparticles compared to clinically utilized Gd-DTPA, and their low r_2_/r_1_ ratio value falling below 2 (1.47 at *T* = 25 °C and 1.31 *T* = 37 °C calculated from Additional file 1: Figure S3) demonstrate their potential to serve as an effective T1 MR imaging contrast agent (Zhang et al. 2018). In addition, the high atomic number of Yb induced enhanced CT signal comparable with iohexol. These results confirm the promise of these nanoparticles in MR/CT multi-modal imaging.

The radiosensitization effect of the ultrasmall NaGdF_4_:Yb50% nanoparticles was then assessed in rat C6 glioma cell line. The survival and the reproductive integrity of the irradiated cells with and without nanoparticle treatment were evaluated through colony formation. One strategy to target the delivery of nanoparticles is to exploit the overexpressed folate receptor, found in many cancer cell lines. C6 cells internalize folic acid-conjugated particles through caveolae-mediated endocytosis (Dong et al. 2014). Taking advantage of the highly expressed folate receptors on C6 glioma cells, nanoparticles with conjugated folic acid (NaGdF_4_:Yb50%-FA) were prepared to improve cellular uptake.

Clonogenic assessment showed increased colony formations with the non-irradiated cells incubated with non-targeted and targeted ultrasmall NaGdF_4_:Yb50% nanoparticles (Fig. 6) in comparison to the untreated control cells. When cells are exposed to environmental stress, such as the presence of nanoparticles, autophagy can be induced as an adaptive response, upregulating expressions of genes and proteins that induce cytoprotection and promote cell survival (Hsu 2012; Tseng and Hsieh 2016; Zabirnyk et al. 2007). Irradiation of cells without nanoparticles did not result in any significant effect on the colony area at 2 Gy dose, suggesting the low intrinsic radiosensitivity of C6 cells (Schueller et al. 2004). Nevertheless, they seemed to form more smaller colonies indicating some effect on their reproductive capacity. On the other hand, both the non-targeted and targeted nanoparticles have clearly shown radiosensitization. These results are in agreement with the recent study investigating the cytotoxicity and radiosensitization of several rare-earth oxide nanoparticles (i.e., Ce, Nd, Gd, and La), wherein Gd_2_O_3_ nanoparticles have shown significant radiosensitization and have generated additional ROS in U-87 MG cell line upon irradiation, without intrinsic toxicity (Lu et al. 2019). As evidenced by a significant difference in the surviving colonies between the non-targeted (NaGdF_4_:Yb50%) and the targeted (NaGdF_4_:Yb50%-FA) nanoparticles at the same concentration, it is imperative that the nanoparticles be associated with the cells to induce effective damage. A new study has shown near complete destruction of tumor spheroids of human ovarian cancer (OVCAR8) when incubated with gadolinium loaded mesoporous silica nanoparticles (Gd-MSN) prior to exposure to monochromatic 50.25 keV X-rays (Matsumoto et al. 2019). It is worth noting that the Gd-MSNs accumulated in the lysosomes located close to the cell nucleus. This highlights the importance not only of the energy compatibility made possible using tunable monochromatic beam radiation, but also by the proximity of the radiosensitizers to the nucleus to destroy the DNA of the tumor cells. This is due to the low energy and consequent short-range characteristics of the Auger electrons from the Gd^3+^ and Yb^3+^ ions in the nanoparticles provide for the possibility of a highly targeted radiation therapy.

In several reported studies, folate-conjugated drug delivery systems have shown significant nuclear uptake (Goren et al. 1949; Porta et al. 2013; Wang et al. 2020). Folic acid-modified silica nanoparticles (FAMSNs) with 100 nm diameter have been observed to accumulate in both the nuclei and the cytoplasm, while unmodified MSNs were found only in the cytoplasm, which confirmed the role of folic acid receptors in the nuclear uptake (Porta et al. 2013). The presence of folic acid receptor α (FRα) in the nuclear membrane has been reported (Boshnjaku et al. 2012; Bozard et al. 2010). It has also been demonstrated that in the presence of folic acid, FRα translocates to the nucleus (Boshnjaku et al. 2012; Mohanty et al. 2017) This mechanism of folic acid is highly compatible in the targeted delivery of radiosensitizers. Combined with the additional multi-modal imaging capabilities of the nanoparticles, localization in the tumor can be ensured prior to irradiation; therefore, the damage to the surrounding normal cells is minimized if not completely prevented. Furthermore, in vitro transmigration assay confirms that both non-targeted and targeted nanoparticle were able to cross the BBB, with the folic acid-modified nanoparticles being 2.4-fold higher. These results further confirm the effectiveness of using folic acid as a target molecule to facilitate transport through BBB.

This study has several limitations. First, although the equimolar ratio of Gd and Yb has shown to achieve the desired properties of CT and MR contrast enhancement and radiosensitization, an optimal ratio between Gd and Yb can only be determined by preparing these hexagonal ultrasmall nanoparticles with different Gd and Yb ratios. Second, in vivo MR and CT imaging still need to be performed to evaluate the efficacy of these nanoparticles as dual contrast agents. Third, the in vivo biodistribution and clearance studies were not performed in GBM-bearing mice to evaluate the percentage and half-life of the nanoparticles that cross the BBB of a diseased animal model. As folate receptors are overexpressed in GBM, it is possible that a higher nanoparticle concentration will be internalized by the brain tumors, which could affect the biodistribution in the brain. A survival study of post-irradiated mice with and without these radiosensitizers have yet to be done to assess their safety and efficacy in vivo.

## Conclusions

A novel, ultrasmall sub-5 nm NaGdF_4_:Yb50% formulation designed to combine imaging and therapy was successfully synthesized and surface modified to render biocompatibility and enhanced cellular uptake. Co-doping of Gd and Yb in equimolar amount allowed the formation of the hexagonal phase of the nanoparticle as well as imparting the nanoparticle with multi-functionality to be used as a bimodal probe for both MR and CT imaging with excellent T1 contrast for MRI and Hounsfield unit (HU) for CT imaging. Bioconjugation of folic acid to the surface of these nanoparticles facilitated BBB crossing and increased cellular uptake to enable efficient radiosensitization effects from the emitted low-energy Auger electrons in brain cancer cells. In vitro radiosensitization experiments in rat C6 glioma cells showed the FA-targeted nanoparticles as very promising radiosensitizers. Hence, these ultrasmall nanoparticles should be further developed to serve as a promising theranostic platform for image-guided radiotherapy.

## Materials and methods

### Materials

Gadolinium chloride hexahydrate (99.999%), ytterbium chloride hexahydrate (99.9%), ammonium fluoride (99.99), sodium hydroxide (97%), oleic acid (90%), 1-octadecene (90%), oleylamine (70%), L-cysteine (97%), diethylenetriaminepentaacetic dianhydride (98%), and H_2_O_2_ (30%) were purchased from Sigma-Aldrich. Methanol (ACS reagent grade, ≥ 99.8%), hexane (ACS reagent grade, ≥ 98.5%), and chloroform (ACS reagent grade, ≥ 99.8%) were purchased from Fisher Scientific. Gadolinium and ytterbium standards for ICP are from Inorganic Ventures and high-purity nitric acid for quantitative trace metal analysis at the ppb level is from BDH Aristar® Plus. All materials were used as received.

### Synthesis

#### Synthesis of ultrasmall α-NaGdF_4_:Yb50%

Ultrasmall nanoparticles were synthesized by modification of a previously reported procedure (Johnson et al. 2011; Li and Zhang 2008). To a 100 mL three-neck flask containing 0.5 mmol of GdCl_3_ ⋅ 6H_2_O and 0.5 mmol of YbCl_3_ ⋅ 6H_2_O were added 9 mL of oleic acid and 15 mL octadecene. The mixture was heated to 160 °C and maintained for 1 h under argon gas with constant stirring and then cooled to room temperature. A solution of methanol (10 mL) containing 4 mmol NH_4_F and 2.5 mmol NaOH was added and the mixture was stirred for 30 min. The temperature is then increased to 100 °C and maintained for 30 min to remove methanol. The solution was then heated at 260 °C for 10 min before cooling to room temperature. The nanoparticles were collected by adding an excess amount of ethanol and centrifuged at 7000 rcf for 5 min. The precipitate was washed with ethanol and finally dispersed in 10 mL hexane for further uses.

#### Synthesis of ultrasmall β-NaGdF_4_:Yb50%

Ultrasmall nanoparticles were synthesized following the procedure described for α-NaGdF_4_:Yb50%, except the solution was stirred for 24 h after the addition of methanol solution (10 mL) containing NH_4_F (4 mmol) and NaOH (2.5 mmol).

#### Synthesis of ultrasmall β-NaGdF_4_

Ultrasmall nanoparticles were synthesized following the procedure described for α-NaGdF_4_:Yb50%, except 1.0 mmol of GdCl_3_ ⋅ 6H_2_O was used.

#### Synthesis of ultrasmall α-NaYbF_4_

6H_2_O, resulted in cubic ultrasmall nanoparticles only.

#### Ligand exchange surface modification

L-Cysteine (60 mg) and diethylenetriaminepentaacetic (DTPA) dianhydride (20 mg) were dissolved in 30 mL H_2_O at pH 9 in a 100 mL round-bottom flask. To this aqueous solution was added 10 mL chloroform solution containing 10 mg of the oleic-capped ultrasmall nanoparticles. The biphasic mixture was stirred vigorously overnight at room temperature to facilitate the transfer of the nanoparticle to the water phase. Excess ligand was removed by twice centrifugation using Vivaspin-20 centrifugal filters (10 kDa MWCO) at 3000 rcf for 15 min and the collected nanoparticles were redispersed in water and filtered through a 0.2 μm syringe filter.

#### Folic acid functionalized ultrasmall nanoparticles (FA-NaGdF_4_:Yb50%)

Five hundred microliters of folic acid dissolved in DMSO (25 mg/mL) in the presence of triethylamine (6.25 μL) was incubated with 6.5 mg of NHS and 6.25 mg of DCC in the dark overnight and then passed through a 0.2 μm filter. The resulting NHS-activated folic acid was then covalently linked to the amino surface of the nanoparticles provided by cysteine ligand by incubating overnight. The resulting NaGdF_4_:Yb50%-FA was centrifuged at 16,000 rcf for 15 min, washed twice and stored in 1 mL H_2_O for future use.

### Characterization

The size and the morphology of the resulting nanoparticles were characterized by transmission electron microscopy (TEM) using a JEM-2010 microscope at an acceleration voltage of 200 kV. The hydrodynamic size was determined using Malvern Zetasizer NanoZS90. Powder X-ray diffraction (XRD) patterns were recorded by a RigakuUltima IV diffractometer, using Cu Kα radiation (λ = 0.15418 nm). The 2θ angle of the XRD spectra was recorded at a scanning rate of 1°/min. Inductively coupled plasma-optical emission spectrometer (ICP-OES) analysis was performed using a Thermo Scientific iCAP 6000 instrument. CT tests were performed on microCTInveon model scanner (Siemens Medical Solutions USA, Inc.). T1 and T2 rates of the nanoparticles were measured on a 4.7 T preclinical MR scanner using increasing concentrations at both 25 °C and 37 °C with an inversion-recovery, balanced steady-state free precession (IR-bSSFP) sequence, and a multiecho CPMG scan, respectively, as described elsewhere. (Dorazio et al. 2011) T1 and T2 relaxivities (mM^−1^ · s^−1^) of the nanoparticles were compared to the commercially available Gd-DTPA contrast agent, Magnevist®.

### Elemental analysis using ICP-OES

Acid digestion was performed by dissolving 0.15 mg of the nanoparticles in 0.5 mL concentrated high-purity HNO_3_ acid overnight and diluting with a 2% HNO_3_ solution to a total volume of 15 mL. The single element standards were prepared with the same acid solution.

### Gd^3+^ ion leaching

The nanoparticles (5 mL, 1 mM Gd) were loaded into a dialysis tubing (Spectrum, 3.5 kD cut-off) and incubated in H_2_O, or DMEM with 10% fetal bovine serum (FBS), or DMEM with 10% FBS supplemented with 10 mM phosphate, at 37 ºC under sink conditions, with rocking for 3 days. The amount of released Gd^3+^ ions in each solution was measured using ICP-OES.

### Biodistribution and clearance

Animal experiments were performed in compliance with guidelines set by the University at Buffalo Institutional Animal Care and Use Committee. Female CD-1 mice were injected intravenously via tail vein with the nanoparticles in 5% dextrose in water at a dose of 2 mg/kg and housed in metabolic cages for 4 days with free access to water and a standard laboratory diet. Urine and feces were collected separately every 4 h and the mice were sacrificed after 96 h through cervical dislocation. Feces and organs including liver, spleen, kidney, brain, heart, and lungs were harvested, frozen, and weighed prior to digestion. The urine, feces, and isolated organs were individually placed in a screw cap polypropylene sample tube and to each were added 3 mL of concentrated nitric acid and 2 mL peroxide (30% by weight) and pre-digested for 24 h. The tubes were then placed in a sonicated water bath for a total of 8 h until the samples were completely dissolved. After digestion, each sample was diluted to 100 mL with a 2% solution of nitric acid. The samples were then passed through a 0.2 μm filter and the Gd content was quantified with inductively coupled plasma mass spectrometry (ICP-MS) utilizing a Thermo Scientific XSERIES 2 ICP-MS Single Quadrupole Mass Spectrometer.

### Cytotoxicity assay

Cell viability was assessed by the PromegaCellTiter 96® AQ_ueous_ One Solution Cell Proliferation (MTS) Assay. C6 cells were seeded into a 96-well flat-bottom microplate (c.a. 10,000 cells/well) at 37 °C and 5% CO_2_ and allowed to attach to the bottom of the microplate overnight. The cells were then treated with different concentrations of NaGdF_4_:Yb50% nanoparticles for 12, 24, and 48 h. After the treatment, the cellular medium was changed to remove the nanoparticles and cell debris, and the AQ_ueous_ One Solution reagent (20 µl/well) was added to the cells and incubated for 4 h. Finally, the absorbance was measured at 490 nm using a microplate reader (Opsys MR microplate reader) to determine the percentage of viable cells in the culture relative to the control wells without nanoparticle treatment.

### Clonogenic assay

Clonogenic assay was performed by growing C6 cells in 6-well plates to 90% confluence and were treated with 100 μg/mL concentration of the nanoparticles overnight. Afterward, cells were irradiated with a 2 Gy X-ray dose using the Faxitron® RX-650 X-ray irradiator at a dose rate of 0.5 Gy/min delivered using 130 kV energy. Plates were then incubated for 4 h at 37 °C in 5% CO_2_, and the cells were subsequently harvested and counted. To assess colony formation, cells were then re-plated at 1000 cells/well in 6-well plates and allowed to form colonies consisting of 50 cells. Colonies were then gently washed with Hank’s Balanced Salt Solution (Gibco® HBSS) and fixed with ice-cold methanol for 10 min, rinsed once again with HBSS, and stained with a 0.5% crystal violet solution for another 10 min. Plates were then rinsed with H_2_O to remove excess stain and were left to dry at room temperature. Images of the plates were then acquired and saved in the tagged image file format (Tiff). The colony area for each plate was then measured using the Colony Area plugin (Guzmán et al. 2014) in ImageJ. Surviving colonies were normalized against control wells without nanoparticle treatment.

### In vitro BBB transmigration assay

We made and validated a cell-based in vitro transwell model of the BBB in our laboratory and used it to examine BBB properties like quantitative permeability and transendothelial migration of nanoparticles. Our 2D in vitro BBB model consists of a two-chamber transwell system in a 12-well culture plate with the upper (luminal) compartment separated from the lower (abluminal) by a semipermeable membrane (polyethylene terephthalate, PET) insert on which the human brain microvascular endothelial cells (BMVECs) were grown to confluency on the upper side, while a confluent layer of normal human astrocytes (NHAs) was grown on the underside. After tight BBB formation was confirmed by the transendothelial electrical resistance (TEER) measurement, the dispersed nanoparticles (100 µg/mL media) were added to the upper chamber (luminal) and incubated at 37 °C in 5% CO_2_. Media from the lower chamber (abluminal) were collected at 1, 5, 24, 48, and 72 h incubation times, and the Gd content was measured using ICP-OES. Percent transmigration was calculated relative to the initial Gd concentration of the media with 100 µg/mL nanoparticles. The TEER was measured again after their crossing of the BBB to make sure that the transmigration was not due to the compromise of BBB.

## Supplementary Information

**Additional file 1.** Additional figures.

## Data Availability

All data generated or analyzed during this study are included in this published article (and its additional information on file).

## Abbreviations

MRI: Magnetic resonance imaging
CT: Computed tomography
RT: Radiotherapy
GBM: Glioblastoma
BBB: Blood–brain barrier
FBS: Fetal bovine serum
DMEM: Dulbecco’s modified Eagle medium
DTPA: Diethylenetriaminepentaacetic
FA: Folic acid
MTS: 3-(4,5,-Dimethylthiazol-2-yl)-5-(3-carboxymethoxyphenyl)-2-(4-sulfophenyl)-2*H*-tetrazolium
