# Supplementary material for: Excretable, ultrasmall hexagonal NaGdF4:Yb50% nanoparticles for bimodal imaging and radiosensitization
Source: Cancer Nanotechnol. 2021 Feb 5;12(1):4. doi: 10.1186/s12645-021-00075-x (PMC7864820; doi:10.1186/s12645-021-00075-x)
Supplement: Supplementary file 1 — Additional file 1. Additional figures. [file 12645_2021_75_MOESM1_ESM.docx]

**Additional Information**

**Excretable, ultrasmall hexagonal NaGdF_4_:Yb50% Nanoparticles for bimodal imaging and radiosensitization**

Jossana A. Damasco^1†^, Tymish Y. Ohulchanskyy^1,6^, Supriya Mahajan^2^, Guanying Chen^1,9^, Ajay Singh^1^, Hilliard L. Kutscher^1,7^, Haoyuan Huang^3^, Steven G. Turowski^4^, Joseph A. Spernyak^4^, Anurag K. Singh^5^, Jonathan F. Lovell^3^, Mukund Seshadri^4,8^, and Paras N. Prasad^1^^[[1]](#footnote-1)^

^1^Department of Chemistry and Institute for Lasers, Photonics and Biophotonics, University at Buffalo, The State University of New York, Buffalo, New York 14260, USA

^2^Department of Medicine, Division of Allergy, Immunology and Rheumatology, University at Buffalo, The State University of New York, New York 14203, USA

^3^Department of Biomedical Engineering, University at Buffalo, The State University of New York, Buffalo, New York 14260, USA

^4^Translational Imaging Shared Resource, Roswell Park Comprehensive Cancer Center, Buffalo, New York 14263, USA

^5^Department of Radiation Medicine, Roswell Park Comprehensive Cancer Center, Buffalo, New York 14263, USA

^6^College of Physics and Optoelectronic Engineering, Shenzhen University, College of Optoelectronic Engineering, 518060 Shenzhen, P.R. China

^7^Department of Anesthesiology, University at Buffalo, The State University of New York, Buffalo, New York 14214, USA

^8^Department of Oral Oncology/Dentistry and Maxillofacial Prosthetics, Roswell Park Comprehensive Cancer Center, Buffalo, New York 14263, USA

^9^School of Chemistry and Chemical Engineering, Harbin Institute of Technology, Harbin, Heilongjiang 15001, P.R. China

^†^Present affiliation: Department of Interventional Radiology, The University of Texas MD Anderson Cancer Center, Houston, TX, 77030, USA

**Supplementary Figures**

**Figure S1** Size distributions from dynamic light scattering (DLS) of hydrophobic NPs in (A) hexane and the (B) surface modified (cysteine/dtpa) NPs in H_2_O indicate good stability and monodispersity in both solvents.


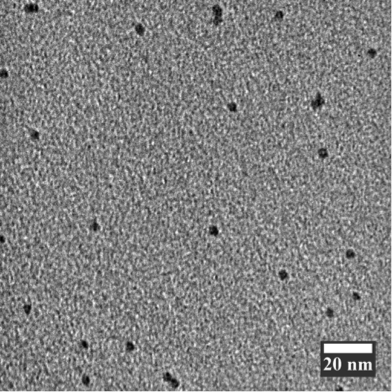


**Figure S2** TEM image of the NPs after ligand exchange also show no aggregation resulting from the process.

**Figure S3** Linear plots of the (a,c) T1 relaxation rates (R1) and (b,d) T2 relaxation rates (R2) as a function of Gd^3+^ concentration to determine the T1 relaxivity (r_1_) and T2 relaxivity (r_2_) of the ultrasmall NP in comparison with Gd-DTPA (Magnevist®) at 25ºC and at 37ºC.

**Figure S4**. Powder X-ray diffraction patterns of resulting ultrasmall (a) β- NaGdF_4_, (b) α-NaGdF_4_:Yb50%, and (c) α-NaYbF_4_ under 30 minutes nucleation time.

**Figure S5** Powder X-ray diffraction patterns of resulting ultrasmall α-NaYbF_4_ synthesized under overnight nucleation time.


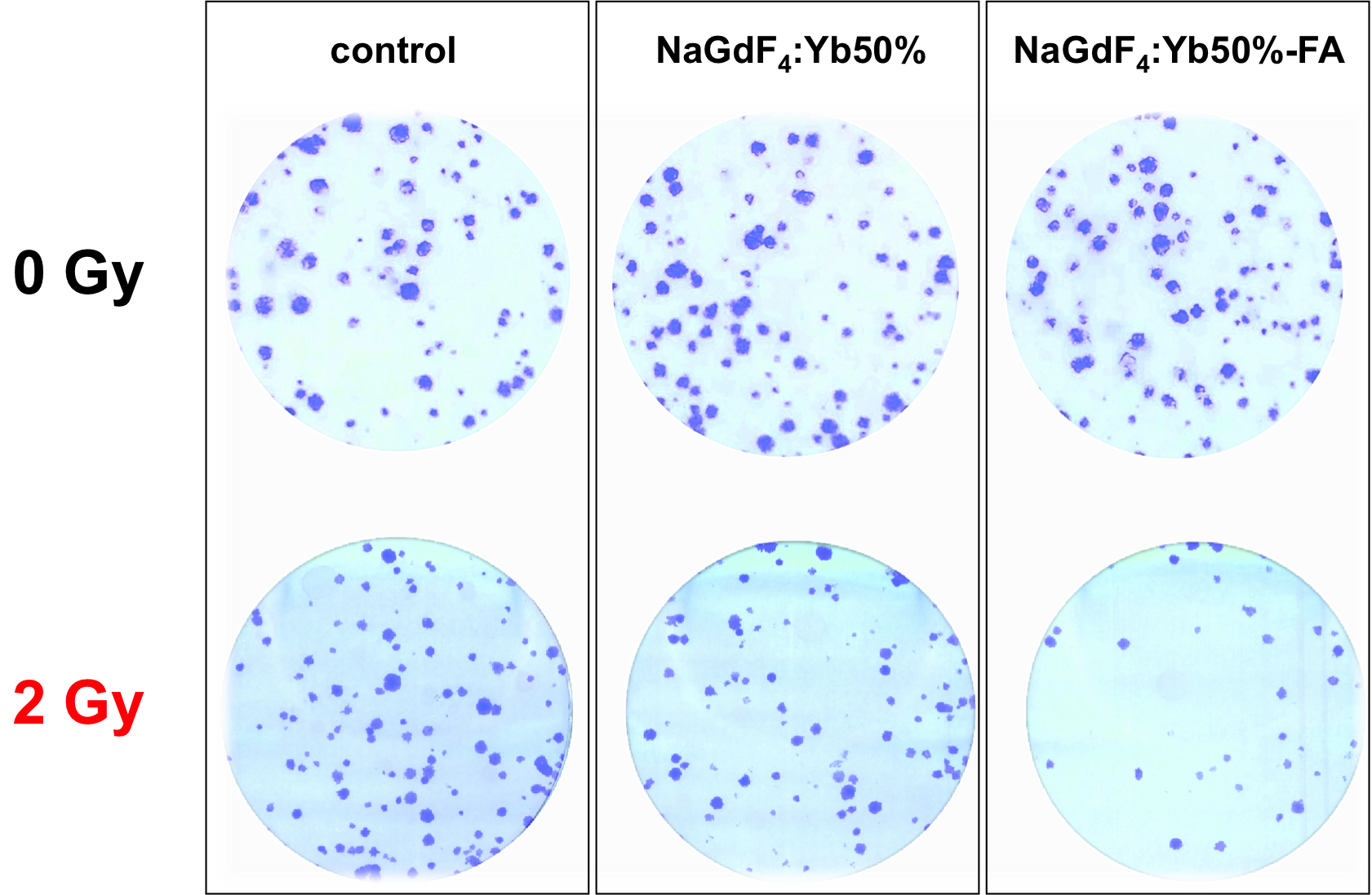


**Figure S6** Colony formation assay of C6 cells


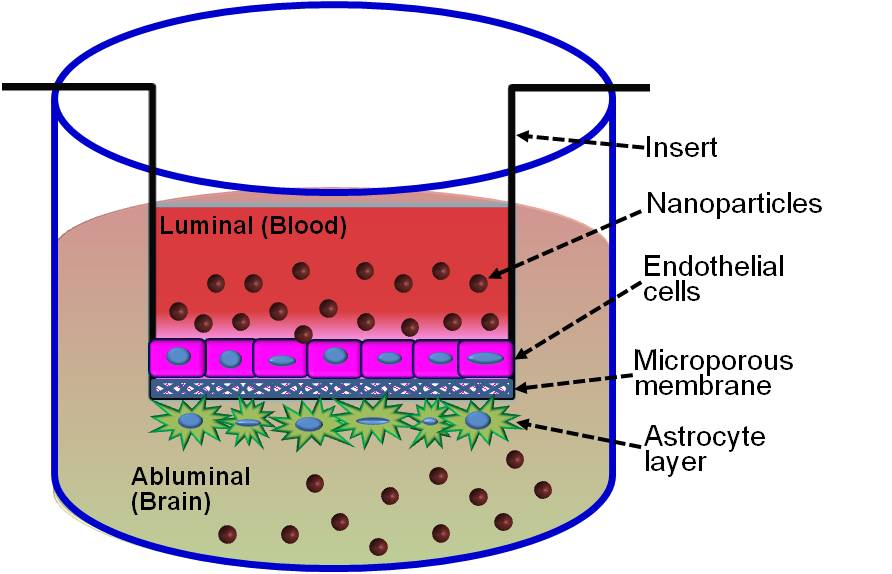


**Figure S7** Schematic diagram of the in vitro BBB model.

1. Corresponding author: pnprasad@buffalo.edu [↑](#footnote-ref-1)
